# Hot Air vs. Vacuum Microwave Drying of Lemon Slices: Effects on Drying Kinetics and Quality Attributes at Different Temperatures

**DOI:** 10.3390/foods15101646

**Published:** 2026-05-09

**Authors:** Hamza Bozkır

**Affiliations:** Food Processing Department, Vocational School of Pamukova, Sakarya University of Applied Sciences, 54900 Pamukova, Türkiye; bozkirhamza@gmail.com

**Keywords:** vacuum microwave drying, hot air drying, lemon slices, drying kinetics, quality attributes

## Abstract

The demand for high-quality dried fruit products with preserved nutritional and sensory attributes has increased significantly in recent years. However, conventional drying methods often result in prolonged processing times and quality degradation. This study compared hot air drying and vacuum microwave drying at different temperatures (50, 60, and 70 °C) in terms of drying kinetics and quality attributes of lemon slices. Drying behavior, moisture content, pH, total titratable acidity, bulk density, total phenolic content, ascorbic acid, and color parameters were evaluated. The drying times for vacuum microwave drying were 144, 108, and 81 min at 50, 60, and 70 °C, respectively, which were substantially shorter than those for hot air drying (360, 204, and 156 min). In addition, vacuum microwave drying resulted in higher drying rates and effective moisture diffusivity. The Page and logarithmic models provided the best fit for the drying kinetics, with higher R^2^ and lower RMSE and χ^2^ values. Furthermore, vacuum microwave drying better preserved quality attributes, with total phenolic content ranging from 120.90 to 174.62 mg GAE/100 g DM and ascorbic acid from 59.87 to 186 mg/100 g DM, consistently higher than in hot air-dried samples. Total titratable acidity was also better retained (3.54–4.63 g citric acid/100 g DM), and lower total color change (ΔE: 14.57–23.23) indicated improved color preservation compared to conventional hot air drying. Overall, vacuum microwave drying offers a promising alternative for producing high-quality dried lemon slices with improved process efficiency.

## 1. Introduction

Drying is one of the oldest and most widely applied methods for food preservation, aiming to reduce the moisture content of food materials to levels that limit microbial growth and slow down chemical and enzymatic reactions. By lowering water activity, drying not only enhances product stability but also reduces weight and volume, thereby decreasing transportation and storage costs [1]. Despite these advantages, the drying process can significantly affect the physicochemical and nutritional quality of food products, particularly when high temperatures and prolonged drying times are involved. Such conditions may lead to the degradation of heat-sensitive bioactive compounds, undesirable changes in color and texture, and reduced rehydration capacity [2,3,4]. These challenges are especially critical for fruits rich in bioactive components, where maintaining quality during drying remains a key concern.

Lemon (*Citrus limon* (L.) *Burm. f.*) is a widely cultivated citrus fruit valued for its distinctive flavor and rich composition of bioactive compounds, including ascorbic acid, citric acid, phenolic compounds, vitamins, and minerals [5]. Owing to these properties, lemon is extensively utilized in a wide range of food products such as juices, concentrates, powders, peel-based ingredients, and dried forms [6]. In particular, dried lemon slices have gained increasing popularity as value-added products used in herbal teas, functional beverages, culinary applications, and as natural flavoring or decorative ingredients in cocktails and gastronomy [7]. Despite these diverse applications, the high moisture content and delicate cellular structure of fresh lemon make it highly perishable and particularly susceptible to quality deterioration during processing. Moreover, key constituents such as vitamin C and phenolic compounds are highly sensitive to heat, oxygen, and prolonged processing conditions, which can result in significant nutritional and sensory losses [8]. Therefore, optimizing drying conditions for lemon is crucial to ensure both product stability and the preservation of its functional and quality attributes.

Conventional hot air (convective) drying is widely used in the food industry due to its simplicity and low cost; however, it is associated with several drawbacks, including long drying times, high energy consumption, and prolonged exposure to heat and oxygen [9]. These conditions often lead to quality deterioration, such as shrinkage, color degradation, loss of volatile compounds, and degradation of heat-sensitive nutrients, as well as limited mass and heat transfer efficiency [10]. To address these limitations, vacuum microwave drying has emerged as a promising alternative. In this technique, microwave energy induces volumetric heating through the interaction with polar molecules, while reduced pressure lowers the boiling point of water, enabling rapid moisture removal at relatively low temperatures [1,11]. This combination accelerates moisture diffusion, shortens drying time, and reduces thermal and oxidative damage [3,4,12,13]. As a result, vacuum microwave drying has been successfully applied to various fruits and vegetables, showing improved drying efficiency and better preservation of quality attributes compared to conventional methods [11,14,15,16,17]. Nevertheless, its performance remains highly dependent on processing conditions, and comparative evaluations with traditional drying methods are still needed for specific products such as lemon.

Previous studies have investigated the drying behavior of lemon slices using various techniques, including vacuum drying, freeze drying, hot air drying, microwave drying, and vacuum infrared drying [18,19,20,21,22]. Despite the well-documented advantages of vacuum microwave drying, its industrial adoption remains limited compared to conventional hot air drying, primarily due to higher equipment costs, operational complexity, and challenges in process control. Therefore, from an industrial perspective, it is essential to determine whether the improvements in drying efficiency and product quality achieved by vacuum microwave drying justify its implementation over simpler and more established hot air systems. In this context, direct and systematic comparisons between these two techniques under comparable conditions are crucial to identify their relative advantages and limitations for specific products such as lemon slices. Therefore, the aim of this study was to comparatively investigate the effects of hot air and vacuum microwave drying at different temperatures (50, 60, and 70 °C) on the drying kinetics and key quality characteristics of lemon slices. This work is expected to contribute to the optimization of drying processes for citrus-based products by providing a better understanding of the relationship between drying method, process conditions, and product quality.

## 2. Materials and Methods

### 2.1. Materials

Fresh Lamas lemons (*Citrus limon* (L.) *Burm. f.*) were obtained from a local market in İzmir, Türkiye, and immediately transported to the laboratory. To ensure uniformity in physical characteristics, lemons of uniform size were selected (average diameter, length, and weight of 57.6 ± 0.6 mm, 78.9 ± 1.8 mm, and 115.0 ± 2.2 g, respectively). Upon arrival, the samples were stored at 4 °C until further processing. Prior to drying, the lemons were thoroughly washed to remove any surface impurities and gently air-dried. Subsequently, the samples were sliced into uniform pieces with a thickness of 4 mm [8] to ensure consistency during the drying experiments. All analytical grade solvents and chemicals were purchased from Merck (Rahway, NJ, USA).

### 2.2. Drying Processes

**Figure 1:**
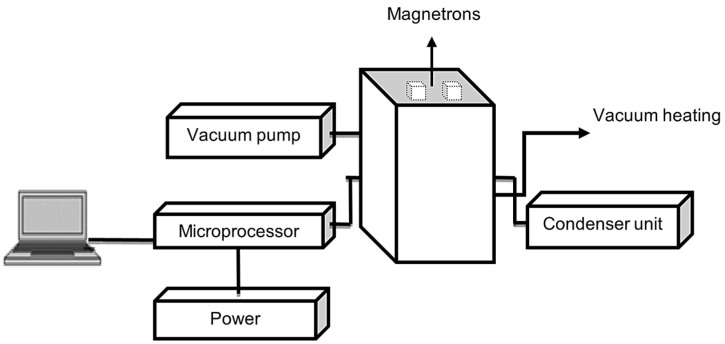
The schematic illustration of vacuum microwave dryer.

Lamas lemon slices, with a total mass of 110 g per batch, were subjected to a drying process using a hot air tray dryer (HD) (Eksis, Isparta, Türkiye) at temperatures of 50, 60, and 70 °C, with an air velocity of 1.8 m/s. The drying of the lemon slices was conducted using a vacuum microwave dryer (VMWD) [12] at temperatures of 50, 60, and 70 °C (877, 801, and 689 mbar vacuum) and a power level of 334 W. The total mass of dried batch lemon slices were 110 g. The schematic structure of vacuum microwave dryer is illustrated in Figure 1. The vacuum microwave dryer is equipped with a temperature control and display system (CT-LT, Optris, Berlin, Germany) and a vacuum control and display system. During drying experiments, the weight loss of the samples was determined at 12 min intervals using a digital balance (Radwag WTC 2000, Radom, Poland) located outside the dryers, until the weight difference was less than 1%. The drying processes were carried out in three replicates under the same conditions. The moisture content was calculated the dry basis of the products during the drying process. This was achieved by means of the following Equation (1) [1]:

(1) $$M_{0}=\frac{W_{0}-W_{K}}{W_{K}}$$

where M_0_ is the initial moisture content of the material on a dry basis (kg H_2_O/kg dry mass (d.m.)); W_0_ is the initial mass of the samples (kg); W_k_ is the dry mass of the samples (kg).

The changes in weight of the dried lemon slice samples over time were monitored, and the dimensionless moisture ratio (MR) and drying rate (DR) values were calculated using Equations (2) and (3) [1].

(2) $$MR=\frac{M_{t}-M_{e}}{M_{i}-M_{e}}$$

(3) $$DR=\frac{M_{t+dt}-M_{t}}{d_{t}}$$

where M_t_, M_i_, M_e_, and M_t+dt_ refer to the moisture content (kg water/kg dm)) at any time t, initial time, equilibrium state and any time t + dt, respectively. t is defined as drying time (min for drying experiments). The value of M_e_ was assumed to be zero, as it is relatively negligible compared to M_i_, following the approach described by Aktas and Baslar [23].

The drying kinetics of the lemon samples were described using the thin-layer drying models listed in Table 1. Model parameters were estimated via non-linear regression analysis using SPSS software (version 20.0; SPSS Inc., Chicago, IL, USA). Following the methodology of Erbay and Icier (2010) [24], the coefficient of determination (R^2^), root mean square error (RMSE) and reduced chi-square (χ^2^) were utilized as the primary statistical criteria to evaluate the goodness-of-fit of the experimental data to the model equations. These models were selected because they encompass a broad range of semi-theoretical and empirical approaches. This selection ensures a robust fit for both the initial and falling-rate drying periods, while facilitating direct comparison with the established literature on sustainable agri-food processing.

The effective moisture diffusivity (D_eff_) was determined based on Fick’s second law of diffusion, as described in Equation (4) [4]:

(4) $$MR=\frac{8}{\pi^{2}}\;\overset{\infty }{\underset{n=1}{\sum }}\frac{1}{{\left(2n-1\right)}^{2}}\;\exp\left[-{\left(2n-1\right)}^{2}\pi^{2}\frac{D_{eff}}{4L^{2}}t\right]$$

where MR is the moisture ratio, L is the half-thickness of the samples (m), D_eff_ is the effective moisture diffusivity (m^2^s^−1^) and t is the drying time (s). For long drying periods, the equation was simplified by considering only the first term of the series, expressed in the following logarithmic form in Equation (5) [4]:


(5) $$\ln\left(MR\right)=ln\left(\frac{8}{\pi^{2}}\right)-\left(\pi^{2}\frac{D_{eff}}{4L^{2}}t\right)$$

The effective moisture diffusivity (D_eff_) was calculated from the slope of the linear relationship between the logarithm of the dimensionless moisture ratio (MR) and drying time, as given in Equation (6) [32]:


(6) $$slope=\pi^{2}\frac{D_{eff}}{4L^{2}}$$

The temperature dependence of the effective moisture diffusivity was determined using the Arrhenius-type equation, as described in Equation (7) [1]:

(7) $$D_{\mathrm{eff}}=D_{0}\;\exp\left(-\frac{E_{a}}{RT}\right)$$

where D_0_ is the pre-exponential constant (m^2^ s^−1^), T is the absolute drying temperature (K), E_a_ is the activation energy (kJ mol^−1^), and R is the universal gas constant (8.314 J mol^−1^ K^−1^). The E_a_ values were determined from the slope of the Arrhenius-type plot of ln (D_eff_) versus 1/T.

### 2.3. Quality Analyses

#### 2.3.1. Moisture Content

The moisture content of lemon slices was determined using a vacuum oven method in accordance with international standard [33]. Approximately 5 g of samples were dried at 70 °C under reduced pressure until a constant weight was achieved. The results were expressed as percentage moisture content on a dry basis.

#### 2.3.2. Bulk Density

The bulk density of the dried lemon slices was determined according to the method described by Goula and Adamopoulos (2005) [34]. A total of 5 g of sample was gently placed into a graduated cylinder of known volume without compaction, and the bulk density was calculated as the ratio of mass to occupied volume. The bulk density was expressed in g/cm^3^.

#### 2.3.3. pH and Total Titratable Acidity (TTA)

A 10 g sample was weighed into an Erlenmeyer flask and homogenized with a small amount of distilled water using a blender. The resulting mixture was transferred to a 100 mL volumetric flask and diluted to volume. After filtration through filter paper, a 25 mL aliquot of the filtrate was collected. The pH of the samples were measured at 20 °C using a calibrated digital pH meter (Thermo Scientific Orion Star, Waltham, MA, USA). Total titratable acidity (TTA) was determined according to standard method by titrating the sample extract with standardized sodium hydroxide solution to a defined endpoint [33]. The results were expressed as milligrams of citric acid per 100 g of dry matter (mg CA/100 g DM).

#### 2.3.4. Total Phenolic Content (TPC)

The total phenolic content of the samples were determined using the Folin–Ciocalteu method as described by Franke et al. (2004) [35], with slight modifications. Briefly, dried samples (2 g) were extracted using 80% (*v*/*v*) methanol under continuous stirring. The extract was centrifuged, and the supernatant was collected for analysis. A 0.5 mL aliquot of the extract was combined with pre-diluted Folin–Ciocalteu reagent and left to react for 5 min. Then, a 7.5% (*w*/*v*) sodium carbonate solution was added, and the mixture was incubated in the dark at room temperature for 30 min. Absorbance was determined at 765 nm using a UV–Vis spectrophotometer (Cary50, Agilent, Santa Clara, CA, USA). Quantification was carried out using a gallic acid calibration curve, and the results were expressed as milligrams of gallic acid equivalents per 100 g of dry matter (mg GAE/100 g DM).

#### 2.3.5. Ascorbic Acid Content

The ascorbic acid content of the samples were determined according to the method described by Hışıl (2004) [36]. Dried lemon samples were extracted using 3% oxalic acid to prevent oxidation. The extract was filtered and analyzed immediately. The determination of ascorbic acid was carried out spectrophotometrically using 2,6-dichlorophenolindophenol (DCPIP) as the reagent. The decrease in absorbance of the DCPIP solution was measured at 520 nm after reaction with the sample extract. A standard ascorbic acid solution was used to construct a calibration curve, and the results were expressed as milligrams of ascorbic acid per 100 g of dry matter (mg/100 g DM).

#### 2.3.6. Color Properties

The color measurements were performed with a Minolta CR-400 colorimeter (Minolta Camera Co., Osaka, Japan), and the results were reported as L* (lightness), a* (redness/greenness), and b* (yellowness/blueness) values. Using the fresh fruit color as a reference (L_0_*, a_0_*, and b_0_*) the total color change (ΔE), chroma (ΔC), and Hue angle were determined using the following Equations (8)–(10):


(8) $$\Delta E=\sqrt{{\left(L^{*}-L_{0}^{*}\right)}^{2}+{\left(a^{*}-a_{0}^{*}\right)}^{2}+{\left(b^{*}-b_{0}^{*}\right)}^{2}}$$

(9) $$C*=\sqrt{{\left(a^{*}-a_{0}^{*}\right)}^{2}+{\left(b^{*}-b_{0}^{*}\right)}^{2}\;\;}$$

(10) $$\mathrm{Hue}\;\mathrm{angle}=tan^{-1}\left(\;\frac{b^{*}}{a^{*}}\;\right)$$

#### 2.3.7. Statistical Analysis

All statistical analyses were carried out using SPSS (version 20.0 for Windows; SPSS Inc., Chicago, IL, USA), where analysis of variance (ANOVA) and Duncan’s multiple range test were applied to identify significant differences between means. Independent samples *t*-tests were employed for comparisons between the two drying groups. Differences were considered statistically significant at *p* < 0.05.

## 3. Results and Discussion

### 3.1. Drying Kinetics

**Figure 2:**
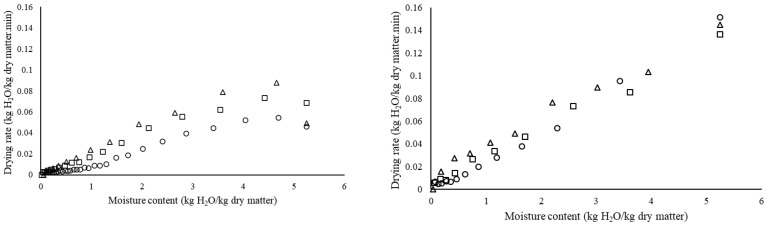
Drying rate vs. moisture content curves for lemon slices under various drying temperatures (○ 50 °C, □ 60 °C, ∆ 70 °C). **Left**; hot air drying, **right**; vacuum microwave drying.

The drying curves of lemon slices at various temperatures for both vacuum microwave and hot drying are illustrated in Figure 2. As expected, increasing the drying temperature led to a marked acceleration in drying rates for both techniques. In addition, the increased temperature during the drying process has been shown to accelerate the drying rate of fruits and vegetables, including apples, melon, pears, and potatoes [37,38,39,40]. In the present study, hot air drying of lemon slices was characterized by three distinct drying periods at 50, 60, and 70 °C. The initial period was characterized by a warming-up phase, followed by a constant-rate period. The subsequent period was marked by a decline in the drying rate toward the end of the process. This observation is consistent with the findings of most studies conducted in this field [21,41]. In contrast, the vacuum microwave drying process was characterized by a falling-rate period across all investigated temperatures, with no apparent constant-rate period. This can be explained by the rapid internal vapor pressure buildup and reduced boiling point inherent to microwave vacuum systems, which bypasses the surface-moisture-dependent constant rate period [42,43]. This observation is consistent with a study by Torki-Harchegani et al. (2016) [44].

The drying rate of vacuum microwave drying was significantly higher than that of hot air drying at all tested temperatures. The drying rates for VMWD were 0.034, 0.044, and 0.058 kg H_2_O/kg d.m. min at 50, 60, and 70 °C, respectively, showing a substantial increase compared to the corresponding HD values of 0.014, 0.024, and 0.031 kg H_2_O/kg d.m. min. These results are consistent with Zielinska et al. (2019) [45], who reported substantially higher drying rates for cranberries under vacuum microwave drying (0.076–0.920 kg H_2_O/kg d.m. min) in comparison with hot air drying (0.023–0.060 kg H_2_O/kg d.m. min at 60–90 °C). Similarly, Bozkir et al. (2021) [4] compared vacuum microwave drying, vacuum infrared drying, and hot air drying for orange peels at 50 °C, reporting that VMWD yielded the highest drying rate among the tested methods. Consistent with these findings, several researchers have reported that vacuum microwave drying significantly accelerates drying rates for various products, including mushrooms, cranberries, moutan cortex, mint leaves, orange slices, and pumpkin slices [1,46,47,48,49,50]. This phenomenon can be attributed to the volumetric heating effect of microwave energy under vacuum conditions, which promotes rapid moisture removal from the product structure.

**Figure 3:**
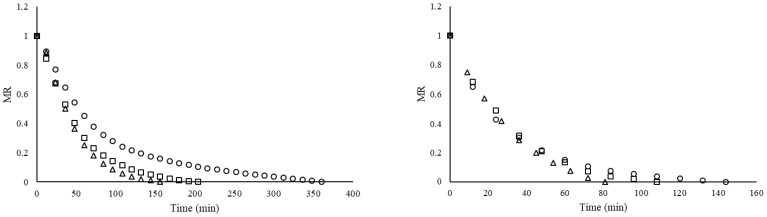
Moisture ratio (MR) curves of lemon slices at various drying temperatures (○ 50 °C, □ 60 °C, ∆ 70 °C). **Left**; hot air drying, **right**; vacuum microwave drying.

The change in dimensionless moisture ratio versus drying time for lemon slices dried at different temperatures using vacuum microwave drying and hot air drying is presented in Figure 3. For both drying methods, an increase in temperature led to a marked reduction in the total drying time required to reach the target moisture content. The drying times for VMWD were 144, 108, and 81 min at 50, 60, and 70 °C, respectively, while the corresponding values for HD were 360, 204, and 156 min. It was determined that VMWD reduced the total drying duration by approximately 53% compared to HD across all investigated temperatures. These results are in close agreement with Zhou et al. (2021) [49], who reported a 54% reduction in drying time of cranberries when using VMWD instead of hot air drying. Consistent with our findings for lemon slices, vacuum microwave drying has been shown to significantly shorten drying time for various products, including orange slices [1], mushrooms [46], pumpkin [47], mint leaves [48], moutan cortex [49], root bark [50], garlic cloves [51], sour cherries [52], banana, grape tomato, carrot slices [53], and cranberries [49,54,55].

### 3.2. Effective Moisture Diffusivity and Activation Energy

The effective moisture diffusivity (D_eff_) values for lemon slices, which represent the rate of internal moisture transfer during the drying process, were determined for both methods (Table 2). The results showed that the D_eff_ values for VMWD at 50, 60, and 70 °C were 7.95 × 10^−10^ 1.05 × 10^−9^, and 1.46 × 10^−9^ m^2^/s, respectively. In comparison, the D_eff_ values for HD at the same temperatures were significantly lower, recorded as 3.89 × 10^−10^, 5.19 × 10^−10^, and 6.49 × 10^−10^ m^2^/s. Previous studies have reported that effective moisture diffusivity values for dried lemon slices ranged from 2.95 × 10^−11^ to 1.90 × 10^−9^ m^2^/s [19,22,36]. In addition, the D_eff_ values obtained in the present study were within the range reported in the literature. The findings indicate that vacuum microwave drying results in enhanced mass transfer and a more efficient drying process compared to hot air drying. Similarly, higher effective moisture diffusivity values have been reported for cranberries, orange slices, mint leaves, and walnuts dried by vacuum microwave drying compared to conventional drying methods [1,45,48,56].

The Arrhenius constant (D_0_) and activation energy (E_a_) were calculated as 1.29 × 10^−6^ m^2^/s and 21.67 kJ/mol (R^2^ = 0.999) for the hot air dryer, and 2.64 × 10^−5^ m^2^/s and 28.00 kJ/mol (R^2^= 0.996) for the vacuum microwave dryer, respectively. Aghbashlo et al. (2008) [57] reported that activation energy values for food materials typically range between 1.27 and 110 kJ/mol. In the present study, the activation energy values calculated for both hot air and vacuum microwave drying were in good agreement with the literature reports. While a high activation energy generally indicates increased sensitivity to temperature variations, the findings of the present study suggest that hot air tray dryer exhibits lower temperature sensitivity. In contrast, the vacuum microwave dryer demonstrated higher sensitivity to temperature changes among the drying systems evaluated. Similarly, Bozkır (2020) [1] reported that hot air and vacuum infrared dryers exhibited the lowest activation energy values, whereas vacuum microwave drying showed the highest values.

### 3.3. Mathematical Modeling

The experimental changes in moisture ratio (MR) for lemon slices, dried via hot air drying and vacuum microwave drying at 50, 60, and 70 °C, are depicted in Figure 3. The empirical MR data were utilized to fit the seven drying models (Diffusion Approach, Henderson and Pabis, Lewis, Logarithmic, Modified Henderson and Pabis, Page, and Wang and Singh) presented in Table 1. The statistical parameters, including model constants, the coefficient of determination (R^2^), root mean square error (RMSE), and reduced chi-square (χ^2^) values for the six distinct models, are summarized in Table 3. In order to provide a comprehensive description of the experimental data, it was necessary to identify the best-fit mathematical model. This was determined based on the highest R^2^ and the minimum root RMSE values, as well as the χ^2^ values [24].

The values for all selected models exceeded 0.902, indicating that they successfully represented the experimental drying behavior. As illustrated in Table 3, the Logarithmic, Page, and Page models provided the highest R^2^ values and the lowest RMSE and χ^2^ values for HD at 50, 60, and 70 °C, respectively. For VMWD, the Page and Logarithmic models yielded the highest R^2^ values (1.00), along with the lowest RMSE (0.00598–0.00705) and χ^2^ (0.000042–0.000071) values across all temperatures. Consequently, this study demonstrates that these models were the most suitable for describing the drying kinetics of lemon slices in a vacuum microwave system. These observations were further supported by the Page, Logarithmic, and Midilli and Kucuk models, which effectively described the drying of lemon slices in hot air, pulsed-vacuum, and microwave-convective dryers [21,44,58]. Consistent with the previous studies, similar results were obtained when the Page and Logarithmic models were utilized to characterize the drying kinetics of orange, mushroom, and carrot slices dried in a vacuum microwave dryer [1,46,59,60].

### 3.4. Impact of VMWD and HD on the Quality Attributes of Lemon Slices

The physicochemical and quality attributes of lemon slices dried via vacuum microwave drying (VMWD) and hot air drying (HD) are summarized in Table 4. Across all drying temperatures (50, 60, and 70 °C), the final moisture content ranged from 6.84 to 7.27 g/100 g d.m. Notably, no statistically significant differences were observed between the two drying techniques regarding final moisture levels (*p* > 0.05). The pH values of the lemon slices increased significantly following the dehydration process, regardless of the drying technique employed, when compared to the raw material. Similarly, an increase in pH values was observed in dried lemon peels, lemon by-products, and peppers relative to the raw material [61,62,63]. The observed pH values for the samples fluctuated between 2.34 and 3.14; moreover, the influence of drying temperature (50, 60, and 70 °C) was statistically significant, as detailed in Table 4 (*p* < 0.05).

Total titratable acidity (TTA) values for lemon slices dried using VMWD and HD at 50, 60, and 70 °C varied from 3.54 to 4.63 g citric acid/100 g dry matter. No statistically significant differences in citric acid content were observed between the 60 and 70 °C groups for lemon slices dried via the VMWD (*p* > 0.05). Conversely, a statistically significant difference was observed between the TTA values of the HD and VMWD groups across all temperatures (*p* < 0.05). The results indicated that lemon slices dried via VMWD consistently exhibited higher average TTA values than those processed using the HD method. Likewise, it was reported that orange slices and lemon peels subjected to vacuum microwave heating demonstrate higher citric acid content in comparison to traditional heating methods [1,62]. Consistent with the literature, our findings confirm that vacuum microwave heating leads to better citric acid retention in citrus samples than traditional thermal treatments.

The results indicate that the total phenolic content (TPC) of fresh lemon slices was 181.53 mg GAE/100 g DM, whereas slices dried using two distinct methods yielded values ranging from 120.90 to 174.62 mg GAE/100 g DM. Our results fall within the range reported in previous studies, where the TPC of dried lemon slices was found to vary between 79 and 335 mg GAE/100 g DM [22,64]. It was observed that both hot air and vacuum microwave drying resulted in a reduction in TPC levels compared to those detected in fresh lemons. This decline was primarily attributable to thermal degradation triggered by heat exposure. These findings were in accordance with previous studies [1,23], which indicated that elevated drying temperatures lead to a reduction in TPC for other citrus fruits, such as mandarin and orange slices. Statistically significant differences were observed between the total phenolic contents (TPC) of lemon slices dried via HD and VMWD across all temperature groups (50, 60, and 70 °C; *p* < 0.05). The results demonstrated that VMWD-dried samples exhibited higher TPC than those dried using the HD method at each tested temperature. Moreover, Leusink et al. (2010) [65], and Wojdyło et al. (2014) [52], Tekgül and Baysal (2018) [62], and Bozkir (2020) [1] reported that the total phenolic content of cranberries, cherries, lemon peel, and orange slices dried via vacuum microwave drying was higher than that of samples dried using hot air dying.

As presented in Table 4, the initial vitamin C content of fresh lemon slices was 208.76 mg a.a./100 g DM. Following the dehydration process, ascorbic acid levels ranged from 59.87 to 186 mg a.a./100 g DM across the two drying methods. These findings align with the previously reported range of 76.40 to 247 mg a.a./100 g DM for dried lemon slices in the literature [8,66]. The vitamin C content of dried lemon slices was found to decrease significantly during drying at 50, 60, and 70 °C in both dryers. In a similar study, Susilo et al. (2022) [66] observed a significant reduction in the ascorbic acid content of lemon slices, which was attributed to the thermal degradation induced by the elevated temperatures (40 to 60 °C) employed during the dehydration process. This reduction is likely due to the thermolabile nature of ascorbic acid, which makes it highly susceptible to thermal degradation and oxidative stress at increased temperatures.

The impact of varying drying temperatures (50, 60, and 70 °C) on the ascorbic acid content of lemon slices was found to be statistically significant (*p* < 0.05) for both vacuum microwave and hot air-drying methods. Notably, lemon slices dried via vacuum microwave drying retained significantly higher vitamin C levels compared to those subjected to hot air drying across all tested temperatures (*p* < 0.05). These results were in agreement with previous studies demonstrating that vacuum microwave drying preserved the vitamin C content of carrots, orange slices, orange peels, and strawberries, more effectively than conventional drying, which can be attributed to the shorter processing times and reduced oxygen exposure inherent in vacuum-assisted microwave systems [1,4,67,68].

The bulk densities of lemon slices dried at different temperatures using different drying techniques are presented in Table 4. The values ranged from 0.48 to 0.90 g/cm^3^, and the drying methods (hot drying and vacuum microwave drying at 50, 60, and 70 °C) had a statistically significant effect on bulk density (*p* < 0.05). The results demonstrated that samples dried by vacuum microwave drying exhibited higher bulk density than those dried by hat air drying at each tested temperature (50, 60, and 70 °C). Moreover, the bulk density of carrot slices [67], lemon peels [62] and orange slices [1] dried by vacuum microwave methods were found to be higher than those of samples dehydrated via conventional hot air drying.

The color parameters (L*, a*, b*, ΔE, C*, and hue angle) of the fresh and dried samples obtained via hot air drying and vacuum microwave drying are presented in Table 4. The L* value, an indicator of lightness, decreased significantly in all dried samples across both drying methods and temperatures (50, 60, and 70 °C) compared to the fresh lemon (*p* < 0.05). Regarding the drying techniques, samples processed via vacuum microwave drying (VMWD) exhibited substantially higher L* values than those subjected to hot air drying. Specifically, L* values for VMWD-treated samples ranged from 72.08 to 74.59, whereas HD samples showed markedly lower values (62.70–69.11). Furthermore, the impact of drying temperature on the L* values of the lemon slices was found to be statistically significant for both drying systems (*p* < 0.05). Both hot air (HD) and vacuum microwave drying (VMWD) methods exerted a statistically significant influence on the a* values of lemon samples across all tested temperatures (50, 60, and 70 °C) (*p* < 0.05). Furthermore, the a* values within the VMWD-treated groups were notably affected by the drying temperature, showing significant differences as the temperature increased (*p* < 0.05). A statistically significant difference was detected in the b* values of lemon slices dried via vacuum microwave drying at 50, 60, and 70 °C (*p* < 0.05). Additionally, the L*, a*, and b* values of the VMWD-dried lemon slices were closer to those of the fresh samples than to the values observed in hot air-dried slices, indicating better color retention. Furthermore, previous studies were reported that the L*, a*, and b* values of various products, including garlic [51], mint leaves [48], sour cherries [52], strawberries [68], and orange slices [1], were better preserved during vacuum microwave drying than during hot air drying.

The total color difference (ΔE) is widely utilized as a key indicator to quantify the overall color change between dried and fresh samples. The ΔE values ranged from 14.57 to 23.23, and both the drying method and temperature (50, 60, and 70 °C) had a significant effect on these values (*p* < 0.05). The highest ΔE values were observed in the hot air-dried samples, whereas the lowest values were exhibited by those subjected to vacuum microwave drying. These results were consistent with previous findings for mint leaves, strawberries, cherries, and orange slices, in which VMWD-treated samples exhibited significantly lower ΔE values than hot air-dried samples [1,48,52,69]. A significant decrease in ΔC values was observed in both drying methods as the temperatures increased from 50 to 70 °C (*p* < 0.05). Furthermore, the variation in hue angle values across the different temperatures was less pronounced in the VMWD samples compared to those dried using the HD. Similar findings were reported, indicating that the ΔC and hue angle values of strawberries [68] and orange slices [1] were better preserved in vacuum microwave-dried samples than in hot air-dried samples (*p* < 0.05).

## 4. Conclusions

The present study systematically compared the drying kinetics and quality attributes of lemon slices subjected to vacuum microwave and hot air drying at different temperatures. The drying times for VMWD were 144, 108, and 81 min at 50, 60, and 70 °C, respectively, which were substantially shorter than those for HD (360, 204, and 156 min). Furthermore, VMWD resulted in higher drying rates (0.034–0.058 kg H_2_O/kg d.m. min.) compared to the corresponding HD values (0.014–0.031 kg H_2_O/kg d.m. min.). Deff values for VMWD at 50, 60, and 70 °C were (7.95 × 10^−10^, 1.05 × 10^−9^, and 1.46 × 10^−9^ m^2^/s, respectively) were higher than those observed for HD (3.89 × 10^−10^, 5.19 × 10^−10^, and 6.49 × 10^−10^ m^2^/s). Kinetic modeling revealed that the Page and logarithmic models most accurately described the drying behavior under both drying systems, confirming their suitability for representing moisture transfer in lemon slices.

Quality evaluations further revealed that drying method and temperature significantly influenced (*p* < 0.05) physicochemical properties, including total titratable acidity, pH, total phenolic content, and ascorbic acid retention. In addition, color analysis (L*, a*, b*, Hue angle, ΔC, and ΔE) indicated that vacuum microwave drying better preserved the visual quality of the product compared to hot air drying, with lower overall color degradation and improved retention of bioactive compounds. Overall, vacuum microwave drying considered to be a more efficient and quality-preserving alternative to conventional hot air drying for lemon slices. These findings highlight its solid potential for industrial application in citrus processing, particularly where both process efficiency and product quality are critical.

## Figures and Tables

**Table 1:** Drying kinetics and mathematical modeling of lemon slices.

Model Names	Model	Reference
Lewis	*MR* = *exp*(−*kt*)	[25]
Page	*Moisture Ratio* = *exp*(−*kt^n^*)	[26]
Henderson and Pabis	*MR* = *aexp*(−*kt*)	[27]
Modified Henderson and Pabis	*MR* = *aexp*(−*kt)* + *aexp*(−*kt*) + *bexp*(−*gt*) + *cexp*(−*ht*)	[28]
Logarithmic	*Moisture Ratio = a.exp*(−*kt*) + *c*	[29]
Diffusion Approach	*MR = aexp*(−*kt*) + (1 − *a*)*exp*(−*kbt*)	[30]
Wang and Singh	*MR* = 1 + *at* + *bt*^2^	[31]

**Table 2:** Effective moisture diffusivity and activation energy for lemon slices.

	Temperature (°C)	D_eff_ (m^2^/s)	R^2^	E_a_ (kJ/mol)
	50	3.89 × 10^−10^	0.989	
HD	60	5.19 × 10^−10^	0.998	21.67 (R^2^ = 0.999)
	70	6.49 × 10^−10^	0.990	
	50	7.95 × 10^−10^	0.994	
VMWD	60	1.05 × 10^−9^	0.988	28.00 (R^2^= 0.996)
	70	1.46 × 10^−9^	0.998	

**Table 3:** Statistical evaluation and comparison of thin layer drying models for lemon slices.

Models	Parameter	HD			VMWD		
		50 °C	60 °C	70 °C	50 °C	60 °C	70 °C
Lewis	*k*	0.012	0.019	0.022	0.033	0.033	0.035
	*X* ^2^	0.00041	0.00052	0.00262	0.00018	0.00042	0.00120
	*RMSE*	0.01991	0.02215	0.04929	0.01304	0.01933	0.03283
	*R* ^2^	0.995	0.995	0.977	0.998	0.996	0.989
Page	*k*	0.015	0.010	0.005	0.045	0.022	0.018
	*n*	0.96	1.149	1.372	0.917	1.104	1.186
	*X* ^2^	0.00035	0.00021	0.00011	0.000042	0.00022	0.00045
	*RMSE*	0.01820	0.01357	0.00990	0.00598	0.01318	0.01902
	*R* ^2^	0.995	0.999	0.999	1	0.998	0.997
Henderson and Pabis	*k*	0.012	0.020	0.023	0.033	0.033	0.036
	*a*	1.004	1.041	1.082	0.984	1.013	1.028
	*X* ^2^	0.00044	0.00036	0.00195	0.00018	0.00044	0.00121
	*RMSE*	0.02020	0.01778	0.04092	0.01229	0.01867	0.03107
	*R* ^2^	0.995	0.996	0.984	0.998	0.997	0.991
Modified Henderson and Pabis	*k*	0.008	0.020	0.023	0.029	0.033	0.036
	*a*	0.348	0.358	0.364	0.439	0.339	0.343
	*g*	0.017	0.020	0.023	0.029	0.033	0.036
	*b*	0.268	0.343	0.365	0.412	0.341	0.342
	*h*	0.017	0.020	0.023	0.102	0.033	0.036
	*c*	0.415	0.340	0.353	0.150	0.333	0.0343
	*X* ^2^	0.00025	0.00047	0.00293	0.00005	0.00087	0.00241
	*RMSE*	0.01427	0.01778	0.04092	0.00537	0.01867	0.03107
	*R* ^2^	0.997	0.996	0.984	1	0.997	0.991
Logarithmic	*k*	0.013	0.019	0.019	0.034	0.029	0.027
	*a*	0.998	1.056	1.137	0.979	1.049	1.128
	*c*	0.021	−0.027	−0.079	0.009	−0.050	−0.127
	*X* ^2^	0.00032	0.00026	0.00116	0.00017	0.000052	0.000071
	*RMSE*	0.01698	0.01473	0.03016	0.01132	0.00604	0.00705
	*R* ^2^	0.996	0.998	0.992	0.999	1	1
Diffusion Approach	*k*	0.015	0.083	0.070	0.102	0.048	0.058
	*a*	0.784	−0.206	−0.804	0.149	−28.023	−40.138
	*b*	−0.452	0.272	0.458	0.286	0.985	0.986
	*X* ^2^	1.3659	0.0000404	0.01827	0.000036	0.00018	0.000044
	*RMSE*	1.1107	0.005806	0.11980	0.00530	0.01118	0.01747
	*R* ^2^	0.997	1	1	1	0.999	0.997
Wang and Singh	*a*	−0.000778	−0.013	−0.019	−0.020	−0.023	−0.026
	b × 10^−3^	0.01489	0.0414	0.0843	0.094	0.127	0.168
	*X* ^2^	0.48754	0.67546	1.01325	0.94093	0.59186	0.30296
	*RMSE*	0.67534	0.77486	0.93193	0.89228	0.68810	0.49231
	*R* ^2^	0.916	0.977	0.902	0.919	0.987	0.996

**Table 4:** Changes in the quality parameters of dried lemon slices.

Moisture Content (g/100 g d.m.)	Fresh	50 °C	60 °C	70 °C
HD	83.99 ± 0.25	7.27 ± 0.20 ^a,A^	7.13 ± 0.10 ^a,A^	7.25 ± 0.13 ^a,A^
VMWD	(g/100 g w.b.)	6.84 ± 0.23 ^a,A^	6.97 ± 0.08 ^a,A^	6.88 ± 0.20 ^a,A^
pH				
HD	2.51 ± 0.02	3.14 ± 0.03 ^a,A^	3.12 ± 0.03 ^a,A^	3.05 ± 0.04 ^a,B^
VMWD		2.34 ± 0.04 ^b,B^	2.49 ± 0.03 ^b,A^	2.50 ± 0.09 ^b,A^
TTA				
HD	5.64 ± 0.01	3.62 ± 0.01 ^b,A^	3.41 ± 0.01 ^b,B^	3.27 ± 0.05 ^b,C^
VMWD		4.83 ± 0.07 ^a,A^	4.63 ± 0.04 ^a,B^	4.50 ± 0.10 ^a,B^
**TPC (mg GAE/100 g d.m.)**				
HD	181.53 ± 0.98	139.23 ± 0.40 ^b,A^	136.31 ± 0.52 ^b,B^	120.90 ± 0.30 ^b,C^
VMWD		174.62 ± 0.10 ^a,A^	170.20 ± 0.40 ^a,B^	165.22 ± 0.70 ^a,C^
**Vitamin C (mg a.a/100 g d.m.)**				
HD	208.76 ± 0.52	87.73 ± 0.61 ^b,A^	64.14 ± 0.50 ^b,B^	59.87 ± 0.31 ^b,C^
VMWD		186.00 ± 1.37 ^a,A^	173.80 ± 1.95 ^a,B^	167.10 ± 1.06 ^a,C^
**Bulk density (g/cm^3^)**				
HD		0.48 ± 0.02 ^b,AB^	0.45 ± 0.04 ^b,A^	0.55 ± 0.05 ^b,A^
VMWD		0.79 ± 0.04 ^a,B^	0.81 ± 0.04 ^a,B^	0.90 ±0.02 ^a,A^
L*				
HD	80.76 ± 0.05	69.11 ± 0.12 ^b,A^	65.60 ± 0.89 ^b,B^	62.70 ± 0.42 ^b,C^
VMWD		72.08 ± 0.42 ^a,C^	73.17 ± 0.23 ^a,B^	74.59 ± 0.34 ^a,A^
a*				
HD	6.45 ± 0.13	4.37 ± 0.13 ^b,A^	4.19 ± 0.04 ^b,A^	3.93 ± 0.15 ^b,B^
VMWD		4.89 ± 0.08 ^a,C^	5.64 ± 0.35 ^a,B^	6.18 ± 0.15 ^a,A^
b*				
HD	21.36 ± 0.10	38.76 ± 0.30 ^a,A^	37.51 ± 0.44 ^a,B^	35.76 ± 0.58 ^a,C^
VMWD		39.03 ± 0.55 ^a,A^	35.46 ± 0.23 ^b,B^	34.56 ± 0.32 ^b,C^
**ΔE**				
HD		21.04 ± 0.27 ^b,C^	22.27 ± 0.16 ^b,B^	23.23 ± 0.13 ^b,A^
VMWD		19.75 ± 0.39 ^a,A^	16.04 ± 0.22 ^a,B^	14.57 ± 0.37 ^a,C^
**ΔC**				
HD		17.52 ± 0.31 ^a,A^	16.31 ± 0.44 ^a,B^	14.61 ± 0.58 ^a,C^
VMWD		17.74 ± 0.56 ^a,A^	14.13 ± 0.21 ^b,B^	13.20 ± 0.37 ^b,C^
**Hue°**				
HD	73.21 ± 0.27	83.57 ± 0.23 ^a,A^	83.62 ± 0.11 ^a,A^	83.72 ± 0.29 ^a,A^
VMWD		82.85 ± 0.18 ^b,A^	80.97 ± 0.49 ^b,B^	79.87 ± 0.22 ^b,C^

Different superscripts (^a,b^) symbolize significant differences within columns (*p* < 0.05). Different superscripts (^A–C^) symbolize significant differences within lines (*p* < 0.05).

## Data Availability

The original contributions presented in this study are included in the article. Further inquiries can be directed to the corresponding author.
